# Enhancement of the Knowledge on Fungal Communities in Directly Brined *Aloreña de Málaga* Green Olive Fermentations by Metabarcoding Analysis

**DOI:** 10.1371/journal.pone.0163135

**Published:** 2016-09-16

**Authors:** Francisco Noé Arroyo-López, Eduardo Medina, Miguel Ángel Ruiz-Bellido, Verónica Romero-Gil, Miguel Montes-Borrego, Blanca B. Landa

**Affiliations:** 1 Food Biotechnology Department, Instituto de la Grasa (IG-CSIC), University Campus Pablo de Olavide, Building 46, Ctra, Utrera, Km 1, 41013, Seville, Spain; 2 Regulatory Council of PDO Aloreña de Málaga table olives, C/ Dehesa, 80, 29560, Pizarra, Malaga, Spain; 3 Crop Protection Department, Institute for Sustainable Agriculture (IAS-CSIC), Avenida Menéndez Pidal s/n Campus Alameda del Obispo, 14004, Cordoba, Spain; Nederlands Instituut voor Ecologie, NETHERLANDS

## Abstract

Nowadays, our knowledge of the fungal biodiversity in fermented vegetables is limited although these microorganisms could have a great influence on the quality and safety of this kind of food. This work uses a metagenetic approach to obtain basic knowledge of the fungal community ecology during the course of fermentation of natural *Aloreña de Málaga* table olives, from reception of raw material to edible fruits. For this purpose, samples of brines and fruits were collected from two industries in Guadalhorce Valley (Málaga, Spain) at different moments of fermentation (0, 7, 30 and 120 days). The physicochemical and microbial counts performed during fermentation showed the typical evolution of this type of processes, mainly dominated by yeasts in apparent absence of *Enterobacteriaceae* and *Lactobacillaceae*. High-throughput barcoded pyrosequencing analysis of ITS1-5.8S-ITS2 region showed a low biodiversity of the fungal community, with the presence at 97% identity of 29 different fungal genera included in 105 operational taxonomic units (OTUs). The most important genera in the raw material at the moment of reception in the industry were *Penicillium*, *Cladosporium*, *Malassezia*, and *Candida*, whilst after 4 months of fermentation in brines *Zygotorulaspora* and *Pichia* were predominant, whereas in fruits were *Candida*, *Penicillium*, *Debaryomyces* and *Saccharomyces*. The fungal genera *Penicillium*, *Pichia*, and *Zygotorulaspora* were shared among the three types of substrates during all the course of fermentation, representing the core fungal population for this table olive specialty. A phylogenetic analysis of the ITS sequences allowed a more accurate assignment of diverse OTUs to *Pichia manshurica*, *Candida parapsilosis/C*. *tropicalis*, *Candida diddensiae*, and *Citeromyces nyonensis* clades. This study highlights the existence of a complex fungal consortium in olive fermentations including phytopathogenic, saprofitic, spoilage and fermentative genera. Insights into the ecology, identification and quantification of fungi species in olive fermentation will facilitate the design of new strategies to improve the quality and safety of this fermented vegetable.

## Introduction

The cultivation of *Olea europaea* tree has a dual purpose, the production of both edible table olives and olive oil, which depends of the olive variety used. When refer table olives, we are talking of a traditional fermented vegetable with many centuries of history in the Mediterranean basin, with a worldwide production which nowadays exceeds 2.5 million tons/year [1]. Green Spanish-style, Greek naturally black and ripe Californian styles are the most popular commercial table olive preparations [2]. However, in the last years, consumers are demanding more traditional and natural homemade seasoned olives. This is the case of *Aloreña de Málaga*, a traditional green olive preparation from Guadalhorce Valley (Málaga, Spain) with a Protected Designation of Origin (PDO) recognized by the European Union [3]. This olive cultivar has unique features which make them quite different from others: its fruits are characterized by an excellent flesh-to-stone ratio, a green–yellow color, a crispy firmness, and a peculiar mild bitter taste. Due to its low-to-moderate concentrations of bitter compounds, the processing does not include alkaline debittering. Thus, they are produced as natural olives and seasoned at the moment of packaging. The manufacturing process is carried out spontaneously by small and medium enterprises placed in, or very close to, the region of production [4].

Lactic acid bacteria (LAB) have an important role during fermentation of lye treated table olives [5]. These microorganisms produce lactic acid and bacteriocins by sugars consumption, contributing to the safe preservation of olives. However, in directly brined (natural) olives, yeasts are also relevant microorganisms coexisting with LAB during fermentation process, or even being the majority microorganisms if LAB are inhibited by the presence of phenolic compounds or the high salt and low pH levels obtained [2, 6]. Yeasts are unicellular eukaryotic microorganisms classified in Fungi kingdom, isolated from many foods and ubiquitous in nature. Their presence during table olive processing was reported in the earliest studies of this product [7–8]. In particular, they can play a double role acting as desirable (due to both technological and probiotic characteristics) or spoilage microorganisms (production of CO_2_, unwanted odors/flavors, the consumption of lactic acid, the softening of fruits or clouding of olive brines) [9]. In the last years, diverse publications have emphasized the great importance that yeasts could have during olive fermentations [9–12].

Recently, diverse molecular methods have been used to identify the yeast species associated to Spanish style [13–14] and natural [11–12, 15–17] industrial olive fermentations. In the specific case of the *Aloreña de Málaga* olive cultivar, diverse authors have used a culture-dependent approach based in RFLP analysis of the 5.8- Internal Transcriber Spacer (ITS) region and sequencing of the D1/D2 domains of 26S rRNA gene to determine the yeast biota associated to this table olive specialty [13, 18–19]. However, the use of methods that rely on the cultivation of microorganism in selective media do not offer a complete profile of the microbial diversity that is present in olive ecosystem and only a small portion of the true microbial population is detected. For this reason, a culture-independent approach (PCR-DGGE) for the study of the yeast biodiversity in *Aloreña de Málaga* fermentations was also used [20]. All these studies were performed exclusively with brines and they did not take into consideration the study of the fungal population adhered to olive surface, which is finally the food intake by consumers.

High-throughput sequencing has emerged as a new culture-independent tool to quantitatively investigate the biodiversity of microbial communities in foods in order to look at dominant as well as minor microbial populations, gaining at the same time information of the fermentative process and the microbiota of raw materials [21–22]. It also has revolutionized the field of food microbial ecology via more accurate identification of microbial taxa without the need for cultivation-dependent methods, showing a huge previously unknown microbial diversity no revealed by conventional methodologies. In the specific case of table olive fermentations, recently this powerful methodology has been used for the study of the bacterial biodiversity adhered to the surface of diverse Italian olive varieties using the 16S rRNA encoding gene as marker [23–24], but no attention was paid in those studies on fungal communities. Unfortunately, information based in high-throughput sequencing of ITS region to determine the fungal population dynamic in fermented vegetables is still scarce. The use of next generation sequencing to decipher a fungal ecosystem requires a different approach, targeting the ITS region, a non-coding DNA sequence situated between the small-subunit ribosomal RNA (rRNA) and large-subunit rRNA genes in the chromosome. The ITS database is somewhat less advanced than for the 16S rDNA gene, but it is gradually improving in the last years [25].

The aim of this study was to use a metagenetic approach to obtain basic knowledge of the changes in the fungal communities through raw material until end of fermentation of PDO *Aloreña de Málaga* table olives, to rationally assess the influence of industry of origin, ecological niche and fermentation time on the population dynamics of these eukaryotic microorganisms. Insight into the fungal life of table olive fermentation will allow us to obtain valuable information of the fermentation process and the structure of fungal community for the design of new strategies to improve the quality and safety of this fermented vegetable.

## Materials and Methods

### Type of samples

Samples were obtained from industrial fermentations of PDO *Aloreña de Málaga* table olives during October 2014 to January 2015. Fruits were harvested at green maturation stage, washed to remove impurities, cracked and directly brined in a 110 g/L NaCl solution in fermentations vessels with 220 L capacity (130 kg fruits). When necessary, fermentation vessels were supplemented with new brine of 120 g/L NaCl and 13 g/L citric acid. Two different industries labelled as COP (UTM ETRS89 coordinate 333969–4066126) and TOL (UTM ETRS89 coordinate 331261–4061750) located at Guadalhorce Valley (Malaga, Spain) were sampled. Both industries are separated by a distance of almost 5.3 km by air but they produce the same denomination of product (traditional PDO *Aloreña de Málaga* olives). Samples were obtained from two different fermentations vessels in each industry from fermentation brines (B) and fruits (F) at the time of reception in the factory (fresh fruit, FF) and after 7 (initial stage of fermentation), 30 (minimum time of brining contemplated by PDO *Aloreña de Málaga* normative) and 120 (moment of packaging established by demand) days of fermentation. Table 1 shows the references of the 28 samples analyzed in the present study and their origin.

**Table 1:** Number of sequences and OTUs analyzed, observed diversity and estimated sample coverage for ITS rRNA amplicons from olives fermentations at two industries.

Sample	Matrix	Industry	Time	Number of reads	Number of OTUs	Good's coverage	Chao1^a^	Richness^a^
FF-COP-0-A	Fresh Fruit	COP	0 months (0 days)	2275	23	99.78	20.03	18.8
FF-COP-0-B	Fresh Fruit	COP	0 months (0 days)	1377	23	99.71	23.33	20.3
F-COP-0-A	Fruit	COP	0 months (7 days)	1516	40	99.67	39.02	35.7
F-COP-0-B	Fruit	COP	0 months (7 days)	1353	32	99.78	32.82	29.5
F-COP-1-A	Fruit	COP	1 month (30 days)	2095	7	99.95	6.20	6.1
F-COP-1-B	Fruit	COP	1 month (30 days)	1933	15	99.64	16.55	10.8
F-COP-4-A	Fruit	COP	4 months (120 days)	2153	30	99.86	29.58	26.0
F-COP-4-B	Fruit	COP	4 months (120 days)	2126	30	99.62	27.00	21.0
B-COP-0-A	Brine	COP	0 months (7 days)	736	50	98.64	57.99	50.0
B-COP-0-B	Brine	COP	0 months (7 days)	853	46	99.06	51.32	44.6
B-COP-1-A	Brine	COP	1 month (30 days)	1603	6	99.94	5.70	5.7
B-COP-1-B	Brine	COP	1 month (30 days)	1584	9	99.81	8.40	7.0
B-COP-4-A	Brine	COP	4 months (120 days)	3303	32	99.82	30.63	25.4
B-COP-4-B	Brine	COP	4 months (120 days)	1790	25	99.83	23.42	22.2
FF-TOL-0-A	Fresh Fruit	TOL	0 months (0 days)	1152	31	98.96	38.87	26.8
FF-TOL-0-B	Fresh Fruit	TOL	0 months (0 days)	1370	26	99.56	27.06	22.7
F-TOL-0-A	Fruit	TOL	0 months (7 days)	1391	22	99.71	21.88	19.9
F-TOL-0-B	Fruit	TOL	0 months (7 days)	1861	28	99.62	23.20	19.9
F-TOL-1-A	Fruit	TOL	1 month (30 days)	923	15	99.57	19.85	14.5
F-TOL-1-B	Fruit	TOL	1 month (30 days)	1725	18	99.65	17.69	14.3
F-TOL-4-A	Fruit	TOL	4 months (120 days)	2511	31	99.76	27.13	21.4
F-TOL-4-B	Fruit	TOL	4 months (120 days)	1822	27	99.73	27.46	22.6
B-TOL-0-A	Brine	TOL	0 months (7 days)	2072	13	99.86	10.39	9.3
B-TOL-0-B	Brine	TOL	0 months (7 days)	1755	48	99.32	48.37	37.7
B-TOL-1-A	Brine	TOL	1 month (30 days)	903	15	99.34	25.30	14.0
B-TOL-1-B	Brine	TOL	1 month (30 days)	1696	14	99.82	15.04	11.1
B-TOL-4-A	Brine	TOL	4 months (120 days)	4186	24	99.83	19.48	15.3
B-TOL-4-B	Brine	TOL	4 months (120 days)	4389	31	99.82	33.80	18.9

^a^ Values were estimated after rarefaction to 730 sequences. A and B stands for the two different fermentation vessels sampled in each industry.

### Monitoring of the industrial fermentations

The analyses of brines for NaCl, pH, titratable and combined acidity were carried out using the routine methods described for table olives [2]. For the counts of microbial populations (*Enterobacteriaceae*, yeasts and LAB) in both brine and fruits, samples were spread in selective media according to methods previously described [26]. Counts were expressed as log_10_ cfu/mL for brines, or log_10_ cfu/g for olives.

### Extraction of DNA from olive samples and pyrosequencing

All samples were treated in the same day for DNA extraction from solid or liquid olive matrix. In the case of brine samples, a volume of 50 mL was taken from fermentation vessels and spun at 9,000 x *g* for 20 min at 5°C. Then, the pellet was washed twice in saline solution (9 g/L NaCl). In the case of fruit samples, 20 g of pulp was homogenized with 50 mL of saline solution in a stomacher and the aqueous phase was spun to get a pellet with same conditions describe above. DNA isolation was done using the PowerFood® Microbial DNA Isolation Kit (MoBio, Carlsbad, Calif.) according to the manufacturer instructions. Purified DNA samples (~10 ng/μL) were stored at –20°C until use.

DNA extracts obtained from the 28 collected samples (4 from FF, 12 from F and 12 from B; see Table 1) were used for the fungal community analysis. This way, the 28 DNA samples were submitted to PCR-amplification of the ITS1-5.8S-ITS2 of rRNA gene. Three independent 20-μL PCRs were performed for each sample using a tailed PCR approach. For the first PCR round the ITS1F (5’-*CTTGGTCATTTAGAGGAAGTAA*-3’) primer that specifically amplify fungal sequences [27] linked to universal M13/pUC forward (5’-*GTTGTAAAACGACGGCCAGT-*3’) sequence and the ITS4 (5’-*TCCTCCGCTTATTGATATGC*-3’) primer linked to universal M13/pUC reverse (5’-*CACAGGAAACAGCTATGACC-*3’) sequence (M13F-*ITS4* and M13R-*ITS1F*) were used [28]. The cycling program for the first round of PCR was an initial denaturation step of 10 min at 95°C, followed by 35 cycles of 1 min denaturation at 95°C, 45 s annealing at 55°C and 1 min extension at 72°C, and a final 10 min extension step at 72°C followed by a 4°C soak. Then, second PCR reactions were performed using a 10x dilution of the first PCR product with the fusion forward primer of the Lib-L consisting of the A-adaptor sequence 5’-*CCATCTCATCCCTGCGTGTCTCCGAC*-3’ followed by the 4-base calibration sequence 5’-*TCAG*-3’, a 10-base MID oligonucleotide to differentiate each of the 28 samples and the 20-base M13F/pUC forward oligonucleotide. The reverse fusion primer consisted of the Lib-L B-adaptor sequence 5’-*CCTATCCCCTGTGTGCCTTGGCAGTC*-3’ followed by the 4-base calibration sequence, and the 20-base M13/pUC reverse oligonucleotide. The cycling program for this second round of PCR was an initial denaturation step of 5 min at 95°C, followed by 15 cycles of 20 s denaturation at 95°C, 20 s annealing at 60°C and 30 s extension at 72°C, and a final 5 min extension step at 72°C followed by a 4°C soak. HPLC-purified oligonucleotides were synthesized by TIB MOLBIOL (Berlin, Germany). All PCR reactions were run in a T100TM Thermal Cycler (Bio-rad, Madrid Spain) using the FastStart High Fidelity Polymerase (Roche Diagnostics GmBH, Mannheim, Germany) and conditions recommended by the manufacturer for pyrosequencing analysis for long amplicons. The PCR products were purified twice with AgencourtH AMPureH XP PCR purification system (Agencourt Bioscience Co., Beverly, MA, USA) and quantified using the Quant-iT dsDNA Assay kit High sensitivity (Invitrogen, Carlsbad, CA, USA) and a fluorometer (BioTek Instruments, Winooski, VT, USA). Subsequently, all samples from each run were pooled in equimolar concentrations and purified again twice with AgencourtH AMPureH XP PCR. Pools of the 28 samples were diluted to obtain a total of 1x10^5^ copies/μL and emulsion PCR was performed with the Lib-L kit (454 Life Sciences) according to manufacturer’s instructions for long reads. DNA positive beads were enriched, counted on the GS Junior Bead Counter, and loaded onto a picotiter plate and run in a 454 Life Sciences (Roche) Junior platform according to the standard platform protocols for long sequencing runs.

### Bioinformatic analysis of pyrosequencing reads

Sequences were processed and analyzed according to procedures previously described [29] using the Quantitative Insights into Microbial Ecology (QIIME) pipeline (version v1.9.1. http://qiime.sourceforge.net/) with default parameters unless otherwise noted. Sequences were first screened for quality using the following parameters: minimum quality score of 25, minimum sequence length of 200 bp, maximum length 1,000 bp, and no ambiguous bases in the entire sequence or mismatches in the primer sequence. Any sequences not meeting these parameters were excluded from downstream analyses. Sequences were then sorted by barcode into their respective samples and the barcode and primer sequences were removed. Chimeras were removed and operational taxonomic units (OTUs) were clustered *de novo* (pick_de_novo_otus.py script) using USEARCH at 97% identity. Taxonomy was assigned to the OTUs against the UNITE version 7 database for ITS sequences [30] available at http://qiime.org/home_static/dataFiles.html and then compared manually to that obtained against NCBI database (last access 25/02/2016). GI identifiers of found best-match sequences were used to extract taxonomy from NCBI taxonomy database. Singleton OTUs were filtered out of the entire dataset to reduce the noise caused by PCR or sequencing error, and we also discarded those OTUs that were present in less than 10% of samples. Sequences are available at the Sequence Read Archive of Genbank under BioProject ID PRJNA317749.

Differences between fungal communities were calculated in QIIME using rarefaction curves of alpha-diversity indexes including estimates of community richness (such as the Chao1 estimator, Richness or the observed number of OTUs present in each sample, and Good’s coverage). Rarefaction analysis was performed using rarefied OTU tables (rarefied to 730 sequences; the lowest number of reads obtained for any of the 28 DNA samples analyzed to control for differing depths of sequencing across the samples), 100 replications, and cut-offs of 97% sequence similarity. Beta-diversity Bray-curtis distance matrices were built after subsampling all samples to an even depth of 730 sequences per sample. Taxonomic abundances within each identified Phylum to genus level were visualized using Krona hierarchical data browser [31]. Principal coordinates analysis (PCoA) was also performed on the Bray-curtis dissimilarity matrices to visualize the differences between the sample types, and visualized using the KiNG graphics program (http://kinemage.biochem.duke.edu/software/king.php). Statistical significance of differences in alpha-diversity were performed with QIIME using a nonparametric two sample t-test with 999 Monte Carlo permutations on number of observations and Chao1 and in beta-diversity a nonparametric ANOSIM tests on Bray-Curtis distance matrices (ITS).

### Phylogenetic analysis

For more accurate assignation of OTUs at species levels, ITS sequences of all OTUs assigned to *Pichia*, *Candida*, *Debaryomyces*, and *Lodderomyces* were aligned using the MEGA software version 5.05 [32] with those acquired from GenBank database for diverse reference type strains of *Candida*, *Pichia*, *Debaryomyces*, *Lodderomyces*, *Citeromyces*, *Wickerhamomyces*, *Yamadazyma*, and *Meyrozyma* species, previously curated in diverse published works of phylogeny and related with table olive processing [9]. Phylogenetic and molecular evolutionary analyses were conducted using the MEGA software version 5.05 only with ITS sequences >300 bp. The evolutionary distance data were calculated from Kimura’s two-parameter model with the maximum-likelihood method [33]. Gaps and missing data were treated as complete deletions. Confidence limits were estimated from bootstrap analysis (1000 replicates).

## Results

### Verification of the fermentation process

The fermentation process of traditional *Aloreña de Málaga* table olives was followed during four months in two different factories by physicochemical and microbiological analyses. The evolution of the main physicochemical characteristics showed a similar behavior in both industries, except salt concentration which was slightly lower in TOL industry at the onset of fermentation (67 g/L) than in COP (80 g/L). The profile of pH and combined acidity in brines was kept practically constant during all fermentation process, with mean values of 4.4 and 0.10 Eq/L, respectively. On the contrary, the salt concentration and titratable acidity increased through fermentation process, reaching similar final values in both industries with 95 g/L and 0.60%, respectively. These data show the acidified and salted environment that olive fermentations represent for microorganisms.

**Fig 1:**
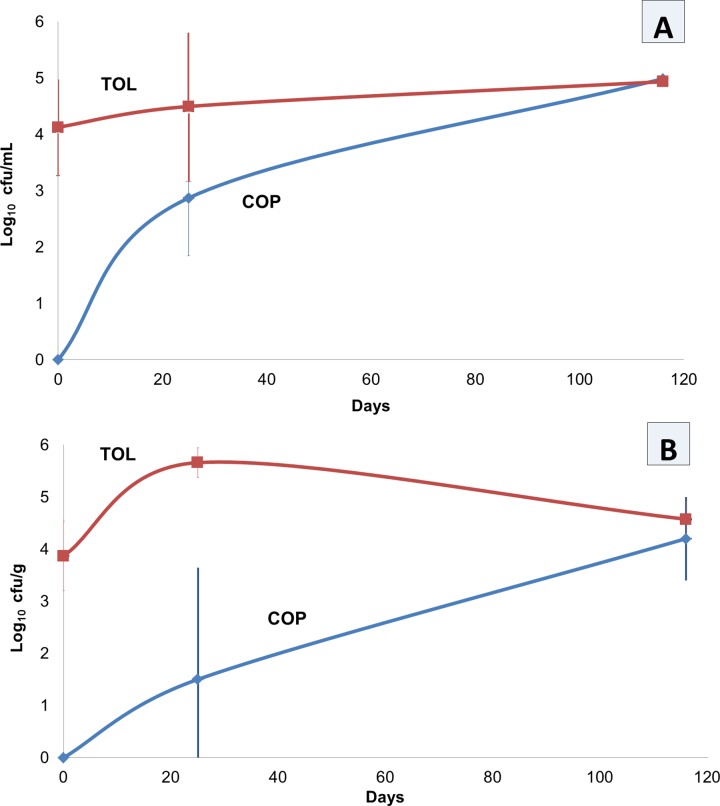
Yeast counts in brines (A) and fruits (B) during industrial fermentation process of *Aloreña de Málaga* table olives. TOL and COP stands for the two different industries analyzed in this work.

Regarding microbial counts, yeasts were the predominant microorganism detected during the study. Thereby, they increased their population levels during fermentation process, with counts higher in TOL than in COP industry for much time of fermentation (Fig 1). However, after four months of study, this fungal group reached practically the same population level in both industries, with 5.0 log_10_ cfu/mL in brines, and 4.5 log_10_ cfu/g in fruits. *Enterobacteriaceae* and LAB were below limit of detection (<1.2 log_10_) during all fermentation process, in both fruits and cover brines.

### Table olive fungal community structure

The complete panel of amplicons of the ITS-PCR products obtained from the 28 samples analyzed yielded a total of 70,983 raw sequences, with a mean of 1,928 reads per sample and average length of 554 bp. After denoising of data for poor quality sequences, we recovered 54,005 high-quality ITS rRNA gene sequences with an average of 1,823 sequences per sample. From those, it was obtained a total of 52,453 sequences that could be appropriately classified into OTUs with a mean of 1,873 classifiable sequences per sample. Table 1 shows the total number of reads obtained in the different samples, as well as the number of OTUs assigned.

According to the analysis of the complete ITS data set, the structure of the global fungi community composition showed big differences between the three types of substrates analyzed (fresh fruit, fermented fruit and brine samples) (S1–S3 Figs). The analysis showed that the fungal phyla *Ascomycota* was the most represented in the three substrates, with 99% of sequences in fermented fruit and brine samples, while the phylum *Basidiomycota* was also represented with 4% of sequences in fresh fruits (with the family *Malasseziaceae*). Within *Ascomycota* phylum, in fresh fruits the classes *Saccharomycetes*, *Dothideomycetes*, and *Eurodomycetes* were practically represented in the same proportions (S1 Fig), whilst in brines and fermented fruit samples the *Saccharomycetes* was clearly the predominant class (S2 and S3 Figs). At family taxa, *Saccharomycetaceae* and *Pichiaceae* were the most important families in both brine and fermented fruit samples. On the contrary, the families *Mycosphaerellaceae* and *Trichocomaceae*, together with *Candida* (included in *Incertae sedis*), were found in higher proportions in fresh fruits (S1–S3 Figs).

**Fig 2:**
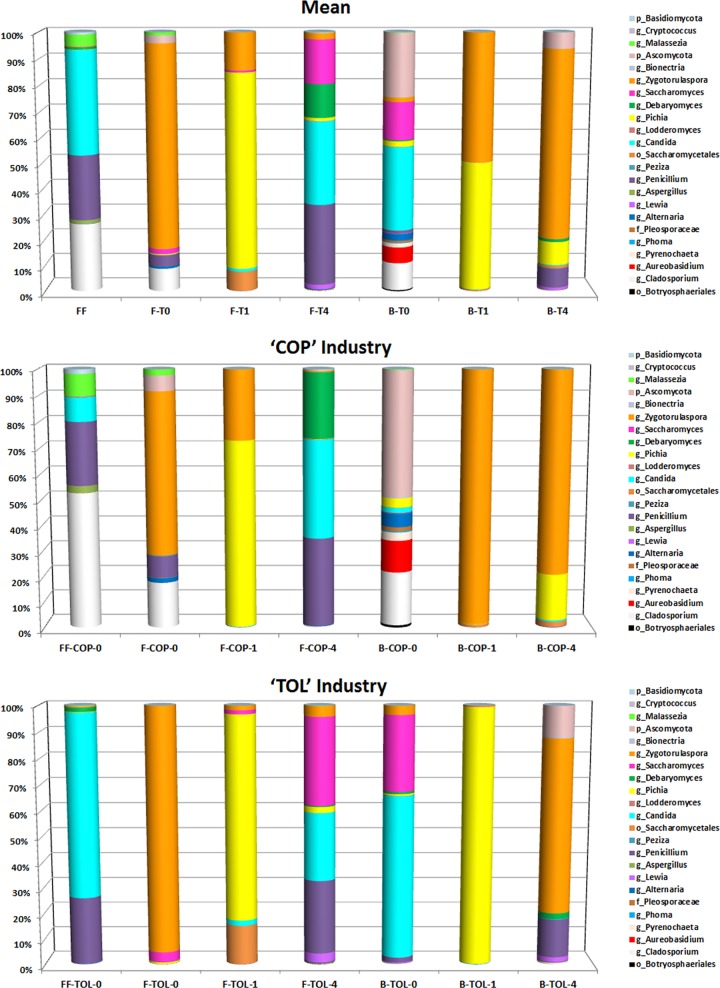
Relative abundance (%) of fungi at genera or family level obtained by pyrosequencing analysis throughout the fermentation process. The different industries (COP and TOL) are shown together (upper graph) and independently (middle and bottom graphs). FF, F, and B stands for fresh fruits, fermented fruits and fermentation brines, respectively, while 0, 1 and 4 stands for the different sampling times (0, 1 and 4 months of fermentation, respectively).

ITS sequences were associated with a total of 105 OTUs belonging to 29 different fungal genera, with an average of 25 observed OTUs (6 to 50) per sample (see Table 1). Only 1.58% of total sequences could not be assigned at genus level. Despite the high number of taxa identified, few genera accounted for most reads. The frequency of fungi genera changed with the type of substrate, during the fermentation process and between factories (Fig 2). This way, the genera *Candida*, *Cladosporium*, *Penicillium*, and *Malassezia* accounted for 95% of sequences in fresh fruits. On the contrary, in the fermented fruits, the majority of genera at the onset of fermentation (7 days) were *Zygotorulaspora* (>75% sequences), while at 30^th^ day were *Pichia* and *Zygotorulaspora*, and at the end of fermentation process (120 days) dominated *Penicillium*, *Candida*, *Saccharomyces*, and *Debaryomyces* were prevalent, in this order. In the fermentation brine samples, at the beginning of fermentation, *Candida*, *Cladosporium*, and *Saccharomyces* were the genera found in higher proportions (>90% sequences), at 30^th^ day were *Zygotorulaspora* and *Pichia*, whilst at the end of fermentation process the dominant genera were *Zygotorulaspora*, *Pichia*, and *Penicillium* (Fig 2). Although there were some differences in the fungal community composition between both industries, those were sown mainly at the end of the fermentation process and in brine samples Fig 2).

### Biodiversity of the fungal community

**Fig 3:**
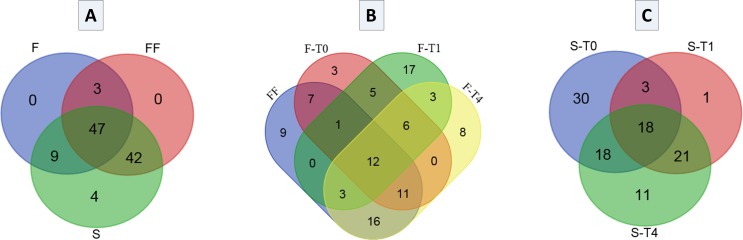
Venn diagrams showing the number of unique and shared OTUs among substrates (A), sampling times in fruits (B) and sampling times in cover brines (C). FF, F, and B stands for fresh fruits, fermented fruits and cover brines, respectively, while 0, 1 and 4 stands for the different sampling times (0, 1 and 4 months of fermentation, respectively).

The Venn diagrams show that the number of unique and shared fungal OTUs changed with the type of substrate and during the course of fermentation (Fig 3). Taking into consideration only the type of substrate, the highest number of fungal OTUs was observed for brines samples (102), followed by fermented olives (92) and fresh fruits (59). A total of 47 OTUs (44.8%) represented the core fungal population for the three types of substrates, whilst fermented fruit and brine samples sharing a higher number of OTUs (89, 85.0%). Only 4 OTUs were unique for brine samples (Fig 3A), belonging to genera *Pyrenochaeta*, *Alternaria*, *Bionectria*, and *Candida* (*C*. *tartivorans*), where there were not specific OTUs for fruit samples. S1 Table shows the OTUs assigned by metabarcoding analysis at genera and species levels shared among the three types of substrates analyzed. Among the fungi species present in all substrates (raw material, fermented fruit and brine samples) we can foreground *Penicillium paneum*, *Aspergillus niger*, *Candida diddensiae*, *Saccharomyces cerevisiae*, *Zygotorulaspora mrakii*, *Debaryomyces hansenii*, and *Lodderomyces elongisporus*, together with genus *Pichia*. Looking exclusively at fruit samples, 12 fungal OTUs (11.9%) were shared by all sampling times including fresh fruits. The number of OTUs increased with fermentation time (i.e., 45 OTUs for F-0, 47 OTUs for F-1, and 59 OTUs for F-4) (Fig 3B). Fermented fruits after 30 days of fermentation (F-1) showed the highest number of unique OTUs (Fig 3B). S2 Table shows the OTUs assigned at genera and species level shared among the fruits in all sampling times. This way, the species *P*. *paneum*, *S*. *cerevisiae*, and *Z*. *mrakii* were present in the fruits during all the course of fermentation, together with genera *Pichia* and *Cladosporium*. In brine samples, a total of 18 fungal OTUs (17.6%) were shared among all times with the brines samples at 7 days of fermentation showing the highest number of total and unique OTUs (Fig 3C). S3 Table shows the OTUs assigned at genera and species levels shared among the brine samples in the different sampling time. *Z*. *mrakii* and *D*. *hansenii* were the only species present in the brine during all the course of fermentation, accompanied by the genera *Pichia* and *Penicillium*. In summary, the fungi genera *Penicillium*, *Pichia*, and *Zygotorulaspora* were shared among the three types of substrates assayed during all the course of fermentation (see S1–S3 Tables), representing the core fungal population for *Aloreña de Málaga* table olive fermentations.

**Fig 4:**
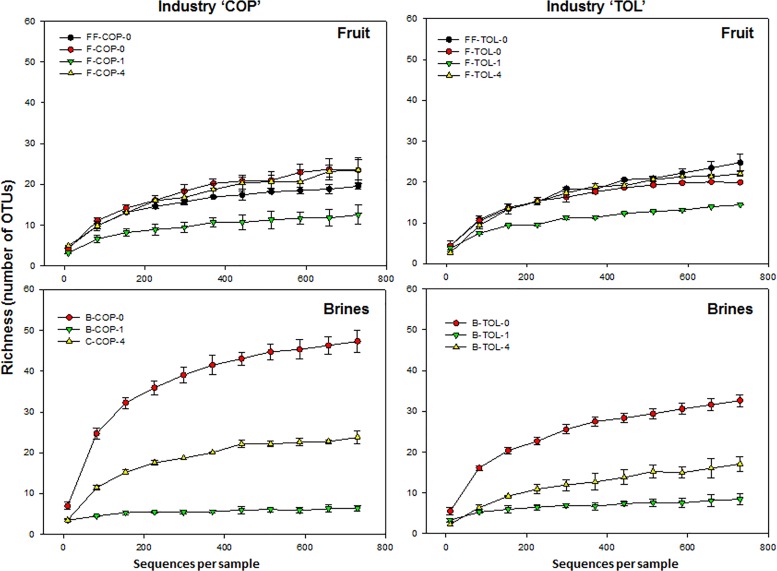
Rarefaction curves of fungal community for the different industries and substrates. FF, F, and B stands for fresh fruits, fermented fruits and cover brines, respectively, while 0, 1 and 4 stands for the different sampling times (0, 1 and 4 months of fermentation, respectively). Data shown are the mean of two fermentation vessels sampled at each industry.

The fungal community was also analyzed using rarefaction curves and richness estimator (Chao1 index). The Chao1 index varied from 5.70 (one of the brine samples obtained from COP industry after 30 days of fermentation) to 57.99 (one of the brine samples obtained from the same factory at the onset of fermentation) (Table 1). The rarefaction analysis assigned to 97% of OTUs similarity showed the achievement of the saturation zone for all samples, suggesting that a number of fungal reads of 730 per sample was satisfactory to obtain a good coverage despite the diversity of sequencing depth between samples (Table 1; Fig 4). Thus, there was a satisfactory coverage of the fungal diversity for all the samples analyzed with Good’s coverage values above 98.6% for all samples (Table 1). Alpha-diversity rarefaction curves indicated that globally there were no significant differences (*P*>0.05) between industries with most differences occurring between fruit and brines samples and during the fermentation process (*P*<0.05), with similar pattern for both alpha-diversity indexes (Chao1 and Richness) (in Fig 4 only data for Richness are shown). For fruit samples, there were no significant (*P*>0.05) differences in alpha-diversity, with a slight trend to decrease alpha-diversity after 30 days of fermentation. For brine samples, there were significant differences (*P*<0.05) during the fermentation process, with the lowest alpha-diversity values occurring 30 days after the fermentation started in both industries and the highest alpha-diversity values at the beginning of fermentation (Fig 4).

**Fig 5:**
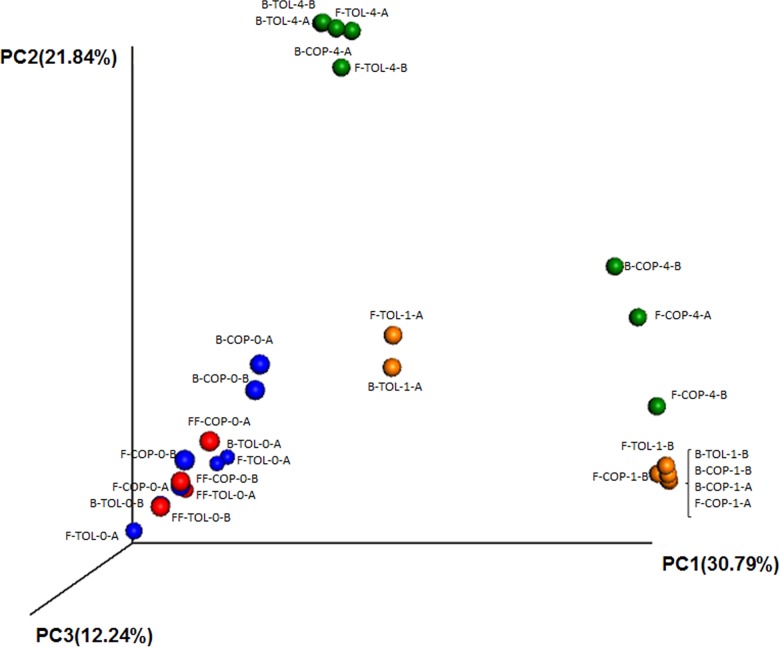
Unweighted UniFrac analysis based in principal coordinates analysis of ITS sequences obtained from different samples. FF, F, and B stands for fresh fruits, fermented fruits and brines, respectively, TOL and COP stands for different industries, 0, 1 and 4 stands for the different sampling times (0, 1, and 4 months of fermentation, in blue, orange and green colors, respectively), while and A and B stands for different fermentation vessels sampled in each industry.

Finally, beta-diversity analysis based in PCoA of Bray-Curtis distance matrices of ITS sequences segregated olive fruits samples unprocessed (FF) and at the beginning of the fermentation process (F-0) from the rest of samples along PC1 (30% of total variance) irrespectively of the industry, while samples at 30^th^ and after 120^th^ days of fermentation were mainly separated along PC2 axis (22% of variance). On the contrary, all fermented fruit and brines samples for both industries tended to group together at 30^th^ (with one exception; sample from fermentation vessel A in TOL industry) while after 120^th^ days of fermentation samples from both industries were clearly differentiated (with one exception; sample B-COP-4-A) pointing out that the changes occurring during the fermentation process (time) were the main drivers of fungal community composition (Fig 5). Thus, ANOSIM test indicated that there were not statistical significant differences (P<0.05) only among the Unweighted UniFrac distances when comparing samples among the different sampling times.

### Phylogenetic assignment of relevant genera

**Fig 6:**
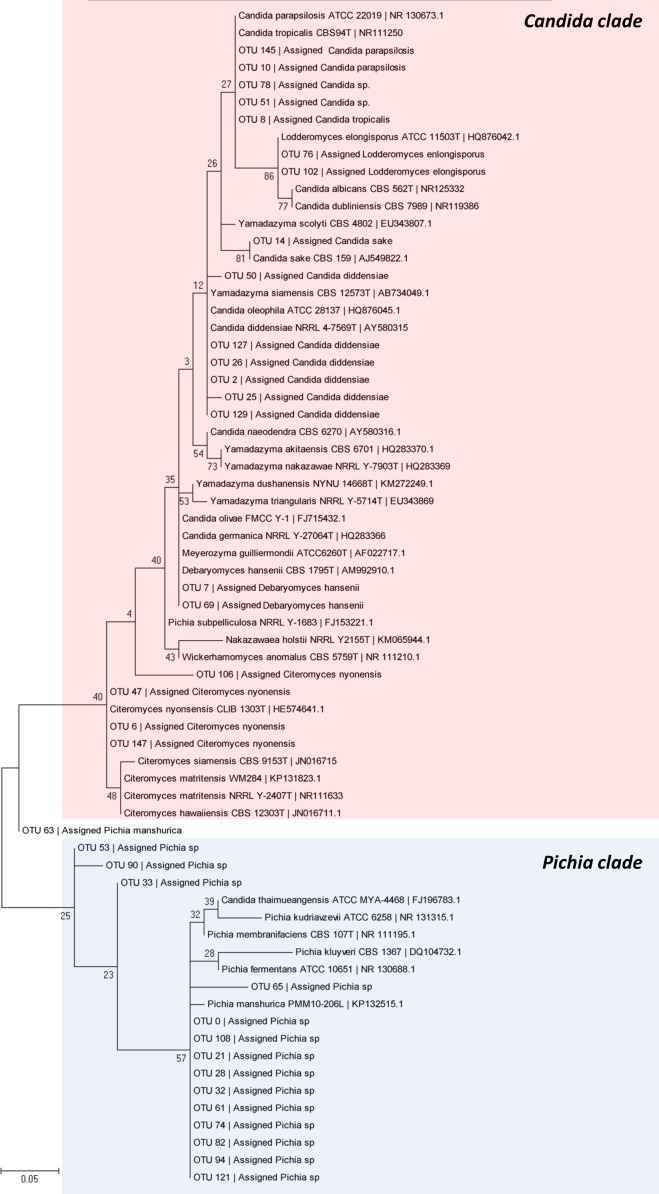
Phylogenetic placement of the OTUs assigned initially by the metabarcoding analysis as *Pichia*, *Candida*, *Debaryomyces*, and *Lodderomyces* genera, respect to diverse type strains of the genera *Candida*, *Pichia*, *Lodderomyces*, *Meyerozyma*, *Wickerhamomyces*, *Debaryomyces*, *Yamadazyma*, and *Citeromyces* related to table olive processing [9]. Their respective GenBank accession numbers are indicated in the phylogenetic tree. The analysis was performed with the ITS sequences and the maximum-likelihood method. Bar, 5 nucleotide changes per 100 nucleotides.

A total of 35 OTUs, assigned initially by the metagenetic analysis as *Candida* spp. (16 OTUs), *Pichia* (15 OTUs), *Debaryomyces* (2 OTUs) and *Lodderomyces* (2 OTUs), were subjected to phylogenetic assignment with the ITS sequences obtained from GenBank for diverse reference type strains of related species. Fig 6 shows the phylogenetic tree obtained after application of maximum-likelihood method with Kimura 2-parameters. Two major clades can be distinguished; one of them included most of the *Pichia* OTUs together with the reference type strains of *Pichia membranifaciens*, *P*. *manshurica*, *P*. *fermentans*, *P*. *kluyveri*, and *P*. *kudriavzevii*. In the case of OTUs 0, 21, 28, 32, 61, 74, 82, 94, 108, and 121, the metagenetic approach was only able to assign them at genus level, but the phylogenetic study showed a close relation of those OTUs with the reference strain of *P*. *manshurica*. On the contrary, the OTUs 33, 53 and 90, albeit were included in the *Pichia* clade confirming the assignation carried out initially against the UNITE database, but could not be closely clustered with any of the type strains of *Pichia* included in the phylogenetic analysis which might indicate they are new taxa (or sequences are not available for comparison in the ITS database). The other large clade was mainly formed by OTUs initially assigned to *Candida*, *Debaryomyces*, and *Lodderomyces*. Thus, OTUs 6, 47, and 147 initially assigned as *Candida nyonensis* were phylogenetically related with the type strains of *Citeromyces nyonensis* (synonymous of *C*. *nyonensis*) and the *Citeromyces* clade. OTUs 8, 10, 51, 78, and 145 were related with the type strains of *C*. *parapsilosis* and *C*. *tropicalis* (OTUs 51 and 78 were only assigned initially as *Candida* spp.), whilst OTUs 2, 25, 26, 127, and 129 were related with the type strain of *C*. *diddensiae*. The closest species to the *Lodderomyces* OTUs, apart from the type strain ATTCC 11503T, were *C*. *albicans* and *C*. *dubliniensis*, whilst the *D*. *hansenii* sequences (anamorph state *C*. *famata*) were related with *Meyerozyma guilliermondii* (anamorph state *C*. *guilliermondi*), *Candida olivae*, and *Candida germanica* type strains. Only OTU 63, initially assigned as *Pichia manshurica*, show a dubious position in the phylogenetic tree. Thus, the phylogenetic analysis confirms many of the initial assignment made against the UNITE database, and also related diverse OTUs that initially were assigned only at genus level with the type strains of certain *Pichia* and *Candida* species.

## Discussion

The main physicochemical and microbiological changes which occurred during fermentation process of *Aloreña de Málaga* olives were related with a slight salt and titratable acidity increase. Both parameters were very similar in both industries and can be considered as the usual ones at the end of the fermentation process of this specialty of natural, cracked, green olives. The flavor and aroma of fermented olives were also tested by a training panel, not detecting the presence of abnormal taste or smells and resulting in the typical product (data not shown). Hence, the samples obtained for metabarcoding analysis can been considered as representative of this type of process, dominated by yeasts because of the high salt and low pH levels obtained [2].

Apart from table olives [23–24], metagenetic analysis has been also used to investigate the changes in bacterial communities in diverse vegetables in brines such as asparagus [34], cucumbers [35], and kimchi [36]. However, not special attention has been paid to the study of fungal communities in vegetables. These microorganisms are especially relevant in directly brined olives due to the inhibition of LAB by the presence of phenolic compounds [5–6, 9]. The only study on this matter was recently carried out by HiSeq Illumina sequencing to determine the fungal communities in serofluid dish, a traditional food in the Chinese culture made from vegetables by fermentation [37]. *Candida* and *Sporpachydermia* were the dominant genera found in that product. Thus, according to our knowledge, there is no available information regarding metagenetic yeast data in the specific case of table olives and vegetables in general. The metagenetic studies of fungal communities in food and beverages are scarce compared to bacteria, with the exception of some products such as fermented yak milk [38], kefir grains and milks [39], kombucha [40], sake [41], cocoa bean [42] and cheese [43] fermentations which all use the ITS as target region.

A great disadvantage of the ITS regions for metabarcoding analysis is related to taxonomical differentiation of phylogenetically related species for some genera that may have similar sequences. Hence, the databases and bioinformatics analysis give reliable microbial identification up to the level of the genus, as occurs in this paper, and they are less confident when used for assignment of fungi to the species level. In addition, a significant part of deposited ITS sequences are not updated or curated, following the latest studies in fungal taxonomy. For this reason, in certain occasions a phylogenetic assignment with reference sequences is performed as a second step for accurate identification [44]. The problematic of differentiating closely related species using short DNA barcodes and pyrosequencing analysis with genus-specific primers was also recently discussed for the oomycete *Phytophthora* [45]. Nevertheless, in our study, this methodology allowed identification of initially assigned OTUs at genus level to *P*. *manshurica* and *C*. *parapsilosis/C*. *tropicalis*, and also the confirmation of the species *C*. *diddensiae*, *D*. *hansenii*, *L*. *elongisporus*, and *C*. *nyonensis*. Our data shows the need and usefulness of this dual approach for accurate and correct identification at species level of relevant fungal genera. However, despite the above limits and biases, the ITS region is widely accepted as the official fungal DNA barcode marker because it can be easily amplified and sequenced by different molecular approaches and provides enough resolution for most fugal species.

Amplicons were analysed with QIIME using a high quality filtering set up in order to minimize the impact of sequencing errors and achieve a reliable identification of fungi population. Despite the high number of taxa identified, few genera accounted for most reads. This way, a conspicuous part of sequences detected were associated with well-known fermentative yeasts. In particular, the genera *Zygotorulaspora* and *Pichia* were found in the raw material, fresh fruits and brines during all the course of fermentation (at least in one of the industries), representing 55.43% of the total of sequences obtained. Thus, they can be considered as the most representative fungi genera of this table olive specialty. The species *Z*. *mrakii* and *P*. *manshurica* were the most important species included in these genera. *Candida* (with 12.3% of total sequences) and *Saccharomyces* (9.13%) were also some genera detected with certain frequency during the fermentation process. The genera *Candida*, *Pichia*, *Zygotorulaspora*, and *Saccharomyces* have been previously described by molecular methods as usual components of the fungal population present during elaboration of *Aloreña de Málaga* [13, 18–20] and other natural table olive elaborations [11–12, 15–17]. Apart from sugar consumption, diverse species of these genera have relevant technological and probiotic characteristics with application in table olive processing, such as production of killer toxins, aromatic compounds, degradation of bitter glucosides, lipase and esterase activities, production of vitamins, biodegradation and bioapsortion of mycotoxins, etc. [9]. The presence of these fermentative yeasts was most habitual during the course of fermentation, except *Candida* spp. which was also detected at high frequencies in the fruits at the moment of reception in the industry.

In our study, the methodology used has also allowed the identification of diverse non-fermentative fungi genera which could play other roles during table olive processing. Some of these genera have been previously described as phytopathogenic microorganisms in olive and other plants, such as *Alternaria*, *Phoma*, *Pyrenochaeta*, and *Bionectria* [44]. However, all them together only represented 0.31% of total sequences, mainly detected at the early stages of fermentation. Thus, their influence on the fermentative process must be scarce. *Cladosporium* and *Aeurobasidium* spp. were also detected in the *Aloreña* samples, mainly in fresh fruits or at the beginning of fermentation, with 5.34% of the total sequences. Both genera were also previously detected by pyrosequencing analysis in leaves, flowers and fruits of olives, suggesting a possible competitive action against the fungal plant pathogens described above [44]. Finally, the study shows also the presence of *Penicillium* (practically in all samples) and *Aspergillus*, both of them considered undesirable microorganisms because of their ability to produce mycotoxins and cellulose and xylanase activities which can produce softening of fruits. Both spoilage genera have been previously described in different table olive processing in presence of oxygen [46–48] and represented the 8.09% of total sequences obtained. *Penicillium* spp. seems to be specially adapted to the fermentative process, because of their presence practically in all samples analyzed.

In summary, results obtained of the present work reveal the complex structure of the fungal community in natural table olive fermentations, from raw material to edible fruits. The fungal consortia showed to contain phytopathogenic, epiphytic, spoilage and fermentative microorganisms that can have a significant impact in the production of this table olive specialty, typically dominated by yeasts. Also, although some differences were found between both industries, the global diversity patters were maintained. We consider that this type of studies are needed to enhance our knowledge of the microbiology of table olive fermentations and fungi in foods. Further studies are also necessary to determine the specific role played by these genera on the quality and safety of table olives.

## Supporting Information

S1 Fig Global taxonomic abundances (%) of fungi community from Phylum to genus level in the fresh fruit samples at the moment of reception in the industry. The different industries and sampling times were considered together for elaboration of the graphs.
(HTML)

S2 Fig Global taxonomic abundances (%) of fungi community from Phylum to genus level in the fermented fruit samples. The different industries and sampling times were considered together for elaboration of the graphs.
(HTML)

S3 Fig Global taxonomic abundances (%) of fungi community from Phylum to genus level in the brine samples. The different industries and sampling times were considered together for elaboration of the graphs.
(HTML)

S1 Table OTUs shared among the three types of substrates (fresh fruits, fermented fruit and brine samples) considering sampling time and industry factors all together. Only OTUs well assigned at genus and species levels by metabarcoding analysis are shown.
(DOC)

S2 Table OTUs shared in fruit samples among all the different sampling time considering the two industries together. Only OTUs well assigned by metabarcoding analysis at genus and species levels are shown.
(DOC)

S3 Table OTUs shared in brine samples among all the different sampling time considering the two industries together. Only OTUs well assigned at genus and species levels by metabarcoding analysis are shown.
(DOC)

## References

[1] IOC (International Olive Oil Council). World table olives figures. 2015. http://www.internationaloliveoil.org/estaticos/view/132-world-table-olive-figures. Last access: May 2015.

[2] Garrido-Fernández A, Fernández-Díez MJ, Adams RM. Table olives: production and processing Chapman & Hall, London. UK 1997.

[3] DOUE. Official Journal of the European Union Regulation N°1068/2012. L318/3. 2012.

[4] López-López A, Garrido-Fernández A. Producción, elaboración, composición y valor nutricional de la Aceituna Aloreña de Málaga Redagua, S.L. Spain 2006.

[5] Hurtado A, Requant C, Bordons A, Rozès N. Lactic acid bacteria from fermented olives. Food Microbiol. 2012; 31:1–8. 22475936 10.1016/j.fm.2012.01.006

[6] Ruiz-Barba JL, Brenes M, Jiménez Díaz R, García P, Garrido A. Inhibition of *Lactobacillus plantarum* by polyphenols extracted from two different kinds of olive brines. J. Appl. Bacteriol. 1993; 74: 15–19.

[7] González Cancho F. Levaduras en la fermentación de aceitunas verdes “estilo español” y su estudio cuantitativo. Grasas Aceites. 1995; 16: 230–234.

[8] Mrak EM, Vaughn RH, Millar MW, Phaff HJ. Yeasts occurring in brines during the fermentation and storage of green olives. Food Technol. 1956; 10: 416–419.

[9] Arroyo-López FN, Romero-Gil V, Bautista-Gallego J, Rodríguez-Gómez F, Jiménez-Díaz R, García-García P, et al Yeasts in table olive processing: Desirable or spoilage microorganisms? Int. J. Food Microbiol. 2012; 160: 42–49. 10.1016/j.ijfoodmicro.2012.08.003 23141644

[10] Bevilacqua A, Corbo MR, Sinigaglia M. Selection of yeasts as starters cultures for table olives: a step-by-step procedure. Frontiers Microbiol. 2012; 3: Art. 194. 10.3389/fmicb.2012.00194 PMC3364525 22666220

[11] Silva T, Reto M, Sol M, Peito A, Peres CM, Peres C, et al Characterization of yeasts from Portuguese brined olives, with a focus on their potentially probiotic behaviour. LWT Food Sci. Technol. 2011; 44: 1349–1354.

[12] Tofalo R, Perpetuini G, Schirone M, Suzzi G, Corsetti A. Yeast biota associated to naturally fermented table olives from different Italian cultivars. Int. J. Food Microbiol. 2013; 161: 203–208. 10.1016/j.ijfoodmicro.2012.12.011 23334098

[13] Bautista-Gallego J, Rodríguez-Gómez F, Barrio E, Querol A, Garrido-Fernández A, Arroyo-López FN. Exploring the yeast biodiversity of green table olive industrial fermentations for technological applications. Int. J. Food Microbiol. 2011; 147: 89–96. 10.1016/j.ijfoodmicro.2011.03.013 21497408

[14] Lucena-Padrós H, Caballero-Guerrero B, Maldonado-Barragán A, Ruiz-Barba JL. Microbial diversity and dynamics of Spanish-style Green table olive fermentations in large manufacturing companies through culture-dependent techniques. Food Microbiol. 2014; 42: 154–165. 10.1016/j.fm.2014.03.020 24929732

[15] Mateus T, Santo D, Saúde C, Pires-Cabral P, Quintas C. The effect of Nacl reduction in the microbiological quality of cracked Green table olives of the Macanilha Algarvia cultivar. Int. J. Food Microbiol. 2016; 218: 57–65. 10.1016/j.ijfoodmicro.2015.11.008 26613162

[16] Nisiotou AA, Chorianopoulos N, Nychas GJE, Panagou EZ. Yeast heterogeneity during spontaneous fermentation of black Conservolea olives in different brine solutions. J. Appl. Microbiol. 2010; 108: 396–405. 10.1111/j.1365-2672.2009.04424.x 20438554

[17] Pereira EL, Ramalhosa E, Borges A, Pereira JA, Baptista P. Yeast dynamics during the natural fermentation process of table olives (*Negrinha de Freixo* cv.). Food Microbiol. 2015; 46: 582–586. 10.1016/j.fm.2014.10.003 25475331

[18] Arroyo-López FN, Durán-Quintana MC, Ruiz-Barba JL, Querol A. Garrido-Fernández A. Use of molecular methods for the identification of yeast associated with table olives. Food Microbiol. 2006; 23: 791–796. 16943084 10.1016/j.fm.2006.02.008

[19] Romero-Gil V, Rodríguez-Gómez F, Garrido-Fernández A, García-García P, Arroyo-López FN. *Lactobacillus pentosus* is the dominant species in spoilt packaged *Aloreña de Málaga* table olives. LWT Food Sci. Technol. 2016; 70: 252–260.

[20] Abriouel H, Benomar N, Lucas R, Gálvez A. Culture-independent study of the diversity of microbial populations in brines during fermentation of naturally fermented Aloreña green table olives. Int. J. Food Microbiol. 2011; 144: 487–496. 10.1016/j.ijfoodmicro.2010.11.006 21122933

[21] Ercolini D. High-throughput sequencing and metagenomics: moving forward in the culture-independent analysis of food microbiology ecology. Appl. Environ. Microbiol. 2013; 79: 3148–3155. 10.1128/AEM.00256-13 23475615 PMC3685257

[22] Kergourlay G, Taminiau B, Daube G, Champomier-Vergès MC. Metagenomic insights into dynamics of microbial communities in food. Int. J. Food Microbiol. 2015; 213: 31–39. 10.1016/j.ijfoodmicro.2015.09.010 26414193

[23] Cocolin L, Alessandria V, Botta C, Gorra R, De Filippis F, Ercolini D, et al NaOH-debittering induces changes in bacterial ecology during table olives fermentation. PLoS One. 2013; 8(7). 10.1371/journal.pone.0069074 PMC3729808 23935928

[24] De Angelis M, Campanella D, Cosmai L, Summo C, Rizzello CG, Caponio F. Microbiota and metabolome of un-started and started Greek-type fermentation of Bella di Cerignola table olives. Food Microbiol. 2015; 52: 18–30. 10.1016/j.fm.2015.06.002 26338113

[25] Santamaria M, Fosso B, Consiglio A, De Caro G, Grillo G, Licciulli F, et al Reference databases for taxonomic assignment in metagenomics. Brief. Bioinform. 2012; 13: 682–695. 10.1093/bib/bbs036 22786784

[26] Rodríguez-Gómez F, Romero-Gil V, Arroyo-López FN, Bautista-Gallego J, García-García P, Garrido-Fernández A. Effect of packaging and storage conditions on microbial survival, physicochemical characteristics and colour of non-thermally preserved Green Spanish-style Manzanilla olives. LWT Food Sci. Technol. 2015; 63: 367–375.

[27] Gardes M, Bruns T. ITS primers with enhanced specificity for basidiomycetes—application to the identification of mycorrhizae and rusts. Mol. Ecol. 1993; 2(2): 113–118. 8180733 10.1111/j.1365-294x.1993.tb00005.x

[28] Gholami M, Bekele WA, Schondelmair J, Snowdon RJ. A tailed PCR procedure for cost-effective, two-order multiplex sequencing of candidate genes in polyploid plants. Plant Biotechnol. J. 2012; 10(6): 635–645. 10.1111/j.1467-7652.2012.00696.x 22489678

[29] Caporaso JG, Kuczynski J, Stombaugh J, Bittinger K, Bushman FD, Costello EK, et al QIIME allows analysis of high-throughput community sequencing data. Nat Methods, 2010; 7(5): 335–336. 10.1038/nmeth.f.303 20383131 PMC3156573

[30] Kõljalg U, Nilsson RH, Abarenkov K, Tedersoo L, Taylor AF, Bahram M, et al Towards a unified paradigm for sequence-based identification of fungi. Mol. Ecol. 2013; 22: 5271–5277. 10.1111/mec.12481 24112409

[31] Ondov BD, Bergman NH, Phillippy AM. Interactive metagenomic visualization in a Web browser. BMC Bioinform. 2011; 12: 385. 10.1186/1471-2105-12-385 PMC3190407 21961884

[32] Tamura K, Peterson D, Peterson N, Stecher G, Nei M, Kumar S. MEGA5: molecular evolutionary genetics analysis using maximum likelihood, evolutionary distance, and maximum parsimony methods. Mol. Biol. Evol. 2011; 28:2731–2739. 10.1093/molbev/msr121 21546353 PMC3203626

[33] Kimura M. A simple method for estimating evolutionary rate of base substitutions through comparative studies of nucleotide sequences. J. Mol. Evol. 1980; 16: 111–120. 7463489 10.1007/BF01731581

[34] Del Árbol JT, Pérez Pulido R, La Storia A, Grande Burgos MJ, Lucas R, Ercolini D, et al Changes in microbial diversity of brined Green asparagus upon treatment with high hydrostatic pressure. Int. J. Food Microbiol. 2016; 216: 1–8. 10.1016/j.ijfoodmicro.2015.09.001 26372734

[35] Medina E, Pérez-Díaz I, Breidt F, Hayes J, Franco W, Butz N, et al Bacterial Ecology of Fermented Cucumber Rising pH Spoilage as Determined by Nonculture-Based Methods. J. Food Sci. 2016; 81: M121–129. 10.1111/1750-3841.13158 26605993 PMC4973622

[36] Jeong SH, Lee HJ, Jung JY, Lee SH, Seo HY, Park WS, et al Effects of red pepper powder on microbial communities and metabolites during kimchi fermentation. Int. J. Food Microbiol. 2013; 160: 252–259. 10.1016/j.ijfoodmicro.2012.10.015 23290232

[37] Chen P, Zhao Y, Zhengrogn W, Liu R, Xu R, Yan L, et al Metagenomic data of fungal internal transcribed spacer from serofluid dish, a traditional Chinese fermented food. Genomics Data. 2016; 7: 134–136. 10.1016/j.gdata.2015.12.028 26981389 PMC4778662

[38] Liu W, Xi X, Sudu Q, Kwok L, Guo Z, Hou Q, et al High-throughput sequencing reveals microbial community diversity of Tibetian naturally fermented yak milk. Annals Microbiol. 2015; 65: 1741–1751.

[39] Marsh AJ, O’Sullivan O, Hill C, Ross RP, Cotter PD. Sequencing based analysis of the bacterial and fungal composition of kefir grains and milks from multiple sources. Plos One. 2013; 7: e69371. 10.1371/journal.pone.0069371 PMC3716650 23894461

[40] Marsh AJ, O’Sullivan O, Hill C, Ross RP, Cotter PD. Sequence-based analysis of the bacterial and fungal compositions of multiple kombucha (tea fungus) samples. Food Microbiol. 2014; 38: 171–178. 10.1016/j.fm.2013.09.003 24290641

[41] Bokulich NA, Ohta M, Lee M, Mills DA. Indigenous bacteria and fungi drive traditional kimoto sake fermentations. Appl. Environ. Microbiol. 2014; 80: 5522–5529. 10.1128/AEM.00663-14 24973064 PMC4136118

[42] Illeghems K, De Vuyst L, Papalexandratou Z, Weckx S. Phylogenetic analysis of a spontaneous cocoa bean fermentation metagenome reveals new insights into its bacterial and fungal community diversity. PLoS One. 2012; 7(5): e38040 10.1371/journal.pone.0038040 22666442 PMC3362557

[43] Wolfe BE, Button JE, Santarelli M, Dutton RJ. Cheese rind communities provide tractable systems for in situ and in vitro studies of microbial diversity. Cell. 2014; 158: 422–443 10.1016/j.cell.2014.05.041 25036636 PMC4222527

[44] Abdelfattah A, Destri Nicosia MG, Cacciola SO, Droby S, Schena L. Metabarcoding analysis of fungal diversity in the phyllosphere and Casposphere of olive (*Olea europaea)*. Plos One. 2015; 10(7): e0131069 10.1371/journal.pone.0131069 26132745 PMC4489200

[45] Prigigallo MA, Abdelfattah A, Cacciola SO, Faedda R, Sanzani M, Cooke DEL, et al Metabarcoding Analysis of *Phytophthora* Diversity Using Genus-Specific Primers and 454 Pyrosequencing. Phytopathol. 2016; 106:305–313. 10.1094/PHYTO-07-15-0167-R 26574783

[46] Baffi MA, Romo-Sánchez S, Ubeda-Iranzo J, Briones-Pérez AI. Fungi isolated from olive ecosystems and screening of their potential biotechnological use. N. Biotechnol. 2012; 29: 451–456. 10.1016/j.nbt.2011.05.004 21689797

[47] Heperkan D, Meric BE, Sismanoglu G, Dalkilic G, Güler FK. Mycobiota, mycotoxigenic fundi, and citrinin production in black olives. Adv. Exp. Med. Biol. 2006: 571: 203–210. 16408603 10.1007/0-387-28391-9_13

[48] Roussos S, Zaouia N, Salih G, Tantaoui-Elaraki A, Lamrani K, Cheheb M, et al Characterization of filamentous fungi isolated from Moroccan olive and olive cake: toxigenic potential of *Aspergillus* strains. Mol. Nutr. Food Res. 2006; 50: 500–506. 16715545 10.1002/mnfr.200600005

