# Supplementary material for: Enhancement of the Knowledge on Fungal Communities in Directly Brined Aloreña de Málaga Green Olive Fermentations by Metabarcoding Analysis
Source: PLoS One. 2016 Sep 16;11(9):e0163135. doi: 10.1371/journal.pone.0163135 (PMC5026345; doi:10.1371/journal.pone.0163135)
Supplement: S1 Fig — The different industries and sampling times were considered together for elaboration of the graphs. (HTML) [file pone.0163135.s001.html]

Javascript must be enabled to view this page.

magnitude
 1.00000000000021
 .947929878386953
 .265944487068435
 0
 0
 0
 .26433555539091
 .26433555539091
 .26433555539091
 .000537694398466
 .000537694398466
 .000537694398466
 1.0712372790585E-03
 0
 0
 .00053561863953
 .00053561863953
 5.356186395285E-04
 .000133904659882
 4.017139796465E-04
 0
 .2653426990608
 .2653426990608
 .2653426990608
 .0137340223008
 .25160867676
 0
 0
 0
 0
 .415839264298423
 .415839264298423
 0
 0
 .402961583601415
 .40269377428165
 2.678093197645E-04
 1.0733130379945E-03
 1.0733130379945E-03
 .011804367659014
 8.4380693315195E-03
 .00095186293174
 2.4144353957545E-03
 0
 0
 0
 0
 .000803427959295
 .000803427959295
 .000803427959295
 .000803427959295
 .05207012161326
 .04350335522761
 .04350335522761
 .04350335522761
 .04350335522761
 0
 0
 0
 0
 .00856676638565
 .00856676638565
 .00856676638565
 .00856676638565
