# Supplementary material for: Enhancement of the Knowledge on Fungal Communities in Directly Brined Aloreña de Málaga Green Olive Fermentations by Metabarcoding Analysis
Source: PLoS One. 2016 Sep 16;11(9):e0163135. doi: 10.1371/journal.pone.0163135 (PMC5026345; doi:10.1371/journal.pone.0163135)
Supplement: S2 Fig — The different industries and sampling times were considered together for elaboration of the graphs. (HTML) [file pone.0163135.s002.html]

Javascript must be enabled to view this page.

magnitude
 .999999999999971
 .995517922059414
 .041053804459891
 7.19010641356667E-05
 7.19010641356667E-05
 7.19010641356667E-05
 3.04804392602673E-02
 3.04804392602673E-02
 3.04804392602673E-02
 6.63217933413333E-05
 6.63217933413333E-05
 6.63217933413333E-05
 1.04351423421467E-02
 0
 0
 0
 0
 1.04351423421467E-02
 .0006855918369
 3.85366324611333E-03
 5.89588725913333E-03
 .119532031172318
 .119532031172318
 .119532031172318
 2.69139722912333E-04
 .119262891449406
 5.25969756738333E-04
 5.25969756738333E-04
 5.25969756738333E-04
 5.25969756738333E-04
 .823942283894493
 .823942283894493
 .025490862138943
 .025490862138943
 .111580625634943
 .111249016668237
 3.31608966706667E-04
 .258586013125787
 .258586013125787
 .42828478299482
 4.30631180968152E-02
 .06548954599265
 .319732118905355
 0
 0
 0
 0
 .010463832775974
 .010463832775974
 .010463832775974
 .010463832775974
 .004482077940557
 .003951503593827
 .003951503593827
 .003951503593827
 .003951503593827
 1.98965380023333E-04
 1.98965380023333E-04
 1.98965380023333E-04
 1.98965380023333E-04
 3.31608966706667E-04
 3.31608966706667E-04
 3.31608966706667E-04
 3.31608966706667E-04
