# Supplementary material for: Enhancement of the Knowledge on Fungal Communities in Directly Brined Aloreña de Málaga Green Olive Fermentations by Metabarcoding Analysis
Source: PLoS One. 2016 Sep 16;11(9):e0163135. doi: 10.1371/journal.pone.0163135 (PMC5026345; doi:10.1371/journal.pone.0163135)
Supplement: S3 Fig — The different industries and sampling times were considered together for elaboration of the graphs. (HTML) [file pone.0163135.s003.html]

Javascript must be enabled to view this page.

magnitude
 .999999999999954
 .99920381723522
 8.07863542573372E-02
 1.49342891278333E-03
 1.49342891278333E-03
 1.49342891278333E-03
 .035935506041185
 .035935506041185
 .035935506041185
 2.07586618876667E-02
 2.07586618876667E-02
 2.07586618876667E-02
 2.25987574157022E-02
 5.96263552616167E-03
 5.96263552616167E-03
 7.46714456391667E-04
 7.46714456391667E-04
 1.58894074331488E-02
 3.06852938950883E-03
 8.99620861248283E-03
 3.82466943115717E-03
 2.88394035928917E-02
 2.88394035928917E-02
 2.88394035928917E-02
 5.32992218313333E-05
 2.87861043710603E-02
 4.26393774651667E-04
 4.26393774651667E-04
 4.26393774651667E-04
 4.26393774651667E-04
 .787230687465687
 .787230687465687
 5.48870633782733E-03
 5.48870633782733E-03
 .10969625880104
 .10936522986325
 .00033102893779
 .20279525348125
 .20279525348125
 .46925046884557
 .005143328651852
 .04898371197112
 .415123428222598
 1.29026144892167E-03
 1.29026144892167E-03
 1.29026144892167E-03
 1.29026144892167E-03
 .10063071669573
 .10063071669573
 .10063071669573
 .10063071669573
 7.96182764734667E-04
 .000448028673835
 .000448028673835
 .000448028673835
 .000448028673835
 1.98811199621333E-04
 1.98811199621333E-04
 1.98811199621333E-04
 1.98811199621333E-04
 1.49342891278333E-04
 1.49342891278333E-04
 1.49342891278333E-04
 1.49342891278333E-04
