# Supplementary material for: Enhancement of the Knowledge on Fungal Communities in Directly Brined Aloreña de Málaga Green Olive Fermentations by Metabarcoding Analysis
Source: PLoS One. 2016 Sep 16;11(9):e0163135. doi: 10.1371/journal.pone.0163135 (PMC5026345; doi:10.1371/journal.pone.0163135)
Supplement: S1 Table — Only OTUs well assigned at genus and species levels by metabarcoding analysis are shown. (DOC) [file pone.0163135.s004.doc]

**S1 Table.** OTUs shared among the three types of substrates (fresh fruits, fermented fruit and brine samples) considering sampling time and industry factors all together. Only OTUs well assigned at genus and species levels by metabarcoding analysis are shown.

| *p_Ascomycota;c_Eurotiomycetes;o_Eurotiales;f_Trichocomaceae; g_Penicillium; s_P. paneum p_Ascomycota;c_Saccharomycetes;o_Saccharomycetales;f_Incertae_sedis;g_Candida; s_C. parapsilosis p_Ascomycota;c_Dothideomycetes;o_Capnodiales;f_Mycosphaerellaceae;g_Cladosporium p_Ascomycota;c_Saccharomycetes;o_Saccharomycetales;f_Incertae_sedis;g_Candida; s_C. diddensiae p_Basidiomycota;c_Malasseziomycetes;o_Malasseziales;f_Malasseziaceae;g_Malassezia p_Ascomycota;c_Saccharomycetes;o_Saccharomycetales;f_Saccharomycetaceae;g_Saccharomyces; s_S. cerevisiae p_Ascomycota;c_Saccharomycetes;o_Saccharomycetales;f_Saccharomycetaceae;g_Zygotorulaspora; s_Z. _mrakii p_Ascomycota;c_Saccharomycetes;o_Saccharomycetales;f_Pichiaceae;g_Pichia p_Ascomycota;c_Saccharomycetes;o_Saccharomycetales;f_Saccharomycetaceae;g_Debaryomyces; s_D. hansenii p_Ascomycota;c_Dothideomycetes;o_Dothideales;f_Dothioraceae;g_Aureobasidium p_Ascomycota;c_Dothideomycetes;o_Pleosporales;f_Pleosporaceae;g_Alternaria p_Ascomycota;c_Saccharomycetes;o_Saccharomycetales;f_Incertae_sedis;g_Lodderomyces; s_L. elongisporus p_Ascomycota;c_Eurotiomycetes;o_Eurotiales;f_Trichocomaceae;g_Aspergillus; s_A. niger* |
| --- |
