# Supplementary material for: Enhancement of the Knowledge on Fungal Communities in Directly Brined Aloreña de Málaga Green Olive Fermentations by Metabarcoding Analysis
Source: PLoS One. 2016 Sep 16;11(9):e0163135. doi: 10.1371/journal.pone.0163135 (PMC5026345; doi:10.1371/journal.pone.0163135)
Supplement: S2 Table — Only OTUs well assigned by metabarcoding analysis at genus and species levels are shown. (DOC) [file pone.0163135.s005.doc]

**S2 Table.** OTUs shared in fruit samples among all the different sampling time considering the two industries together. Only OTUs well assigned by metabarcoding analysis at genus and species levels are shown.

| *p_Ascomycota;c_Eurotiomycetes;o_Eurotiales;f_Trichocomaceae;g_Penicillium; s_P. paneum p_Ascomycota;c_Saccharomycetes;o_Saccharomycetales;f_Saccharomycetaceae;g_Saccharomyces;s_S. cerevisiae p_Ascomycota;c_Saccharomycetes;o_Saccharomycetales;f_Saccharomycetaceae;g_Zygotorulaspora; s_Z. mrakii p_Ascomycota;c_Saccharomycetes;o_Saccharomycetales;f_Pichiaceae;g_Pichia p_Ascomycota;c_Dothideomycetes;o_Capnodiales;f_Mycosphaerellaceae;g_Cladosporium p_Ascomycota;c_Eurotiomycetes;o_Eurotiales;f_Trichocomaceae;g_Penicillium* |
| --- |
