# Supplementary material for: Enhancement of the Knowledge on Fungal Communities in Directly Brined Aloreña de Málaga Green Olive Fermentations by Metabarcoding Analysis
Source: PLoS One. 2016 Sep 16;11(9):e0163135. doi: 10.1371/journal.pone.0163135 (PMC5026345; doi:10.1371/journal.pone.0163135)
Supplement: S3 Table — Only OTUs well assigned at genus and species levels by metabarcoding analysis are shown. (DOC) [file pone.0163135.s006.doc]

**S3 Table.** OTUs shared in brine samples among all the different sampling time considering the two industries together. Only OTUs well assigned at genus and species levels by metabarcoding analysis are shown.

| *p_Ascomycota;c_Saccharomycetes;o_Saccharomycetales;f_Pichiaceae;g_Pichia p_Ascomycota;c_Saccharomycetes;o_Saccharomycetales;f_Saccharomycetaceae;g_Zygotorulaspora; s_Z. mrakii p_Ascomycota;c_Saccharomycetes;o_Saccharomycetales;f_Saccharomycetaceae;g_Debaryomyces; s_D. hansenii p_Ascomycota;c_Eurotiomycetes;o_Eurotiales;f_Trichocomaceae;g_Penicillium* |
| --- |
